# Gasdermin C sensitizes tumor cells to PARP inhibitor therapy in cancer models

**DOI:** 10.1172/JCI166841

**Published:** 2024-01-02

**Authors:** Shuanglian Wang, Chiung-Wen Chang, Juan Huang, Shan Zeng, Xin Zhang, Mien-Chie Hung, Junwei Hou

**Affiliations:** 1 Department of Otolaryngology Head and Neck Surgery,; 2 Xiangya Cancer Center, and; 3 Center for Molecular Oncology and Immunology, Xiangya Hospital, Central South University, Changsha, Hunan, China.; 4 Otolaryngology Major Disease Research Key Laboratory of Hunan Province, Changsha, Hunan, China.; 5 Clinical Research Center for Pharyngolaryngeal Diseases and Voice Disorders in Hunan Province, Changsha, Hunan, China.; 6 National Clinical Research Center for Geriatric Disorders, Xiangya Hospital, Central South University, Changsha, Hunan, China.; 7 Institute of Biological Chemistry, Academia Sinica, Taipei, Taiwan.; 8 Department of General Surgery, Xiangya Hospital, Central South University, Clinical Research Center For Breast Cancer in Hunan Province, Changsha, Hunan, China.; 9 Department of Oncology, Xiangya Hospital, Central South University, Changsha, Hunan, China.; 10 Graduate Institute of Biomedical Sciences, Institute of Biochemistry and Molecular Biology, Research Center for Cancer Biology, Cancer Biology and Precision Therapeutics Center, and Center for Molecular Medicine, China Medical University, Taichung, Taiwan.; 11 Department of Biotechnology, Asia University, Taichung, Taiwan.

**Keywords:** Oncology, Therapeutics, Cancer, Drug therapy, T cells

## Abstract

Several poly (ADP-ribose) polymerase (PARP) inhibitors (PARPi) are approved by FDA to treat cancer with BRCA mutations. BRCA mutations are considered to fuel a PARPi killing effect by inducing apoptosis. However, resistance to PARPi is frequently observed in the clinic due to an incomplete understanding on the molecular basis of PARPi function and a lack of good markers, beyond BRCA mutations, to predict response. Here, we show that gasdermin C (GSDMC) sensitized tumor cells to PARPi in vitro and in immunocompetent mice and caused durable tumor regression in an immune-dependent manner. A high expression level of GSDMC predicted better response to PARPi treatment in patients with triple-negative breast cancer (TNBC). PARPi treatment triggered GSDMC/caspase-8–mediated cancer cell pyroptosis (CCP) that enhanced PARPi killing of tumor cells. GSDMC-mediated CCP increased memory CD8^+^ T cell population in lymph node (LN), spleen, and tumor and, thus, promoted cytotoxic CD8^+^ T cell infiltration in the tumor microenvironment. T cell–derived granzyme B (GZMB) activated caspase-6, which subsequently cleaved GSDMC to induce pyroptosis. Interestingly, IFN-γ induced GSDMC expression, which, in turn, enhanced the cytotoxicity of PARPi and T cells. Importantly, GSDMC promoted tumor clearance independent of BRCA deficiency in multiple cancer types with PARPi treatment. This study identifies a general marker and target for PARPi therapy and offers insights into the mechanism of PARPi function.

## Abstract 2

Gasdermin C serves as a general marker for PARP-inhibitor therapy in cancers regardless of BRCA status through pyroptosis-augmented antitumor immunity.

## Graphical abstract

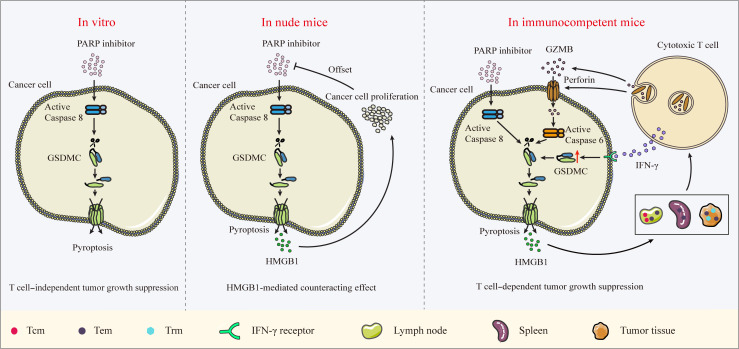

## Introduction

Poly (ADP-ribose) polymerase inhibitors (PARPi) have been emerging as promising therapeutics for many diseases, including cancer (1). Niraparib, rucaparib, and olaparib have been approved for treatment of patients with BRCA-mutant ovarian cancer, prostate cancer, fallopian tube cancer, and peritoneal cancer (2, 3). Additionally, talazoparib and olaparib have been approved for BRCA-mutant breast cancer (2, 3). Mechanistically, PARPi inhibits PARP catalytic activity, inducing ‘trapping’ of PARP in a complex with a DNA strand, which results in overwhelming genomic instability in HR-deficient tumors — those with genetic BRCA1/2 mutations — to induce apoptotic cell death (4). However, the cytotoxicity and efficacy of PARPi currently used in clinic or in ongoing clinical trials, including niraparib, rucaparib, talazoparib, olaparib, and veliparib, in contrast with their abilities of PARP activity inhibition and trapping, seem to have a much broader range (4, 5) of functions, which raises the important questions of whether there exist other molecular mechanisms of PARPi function in tumor cells and how to increase the sensitivity of cancer to PARPi.

Olaparib could increase death ligand-induced caspase-8 cleavage in cancer cells (6) and modulate pyroptosis in the mouse model of Huntington’s disease (7). Our recent study showed that gasdermin C (GSDMC) cleavage by caspase-8 switched apoptosis to pyroptosis in triple-negative breast cancer (TNBC) and other cancer types (8), indicating that olaparib may trigger pyroptosis in GSDMC-positive TNBC cells. PARPi has been reported to boost T cell–dependent antitumor immunity via intratumoral STING pathway activation (9, 10) and drive immunogenic cell death (ICD) in response to IFN-γ in the tumor microenvironment (11). Considering the late observations that cancer cell pyroptosis (CCP), as a form of ICD, suppressed tumor growth by activating antitumor immunity (12–14), we asked whether the CCP-induced antitumor immunity by GSDMC might sensitize tumor cells to PARPi treatment. And we found that GSDMC increased PARPi sensitivity in multiple cancer types through expansion of memory CD8^+^ T cells in lymphoid tissue and tumors, suggesting the favorable role of CCP in PARPi therapy for cancer.

## Results

### GSDMC enhanced the efficacy of PARPi in an immune-dependent manner.

**Figure 1:**
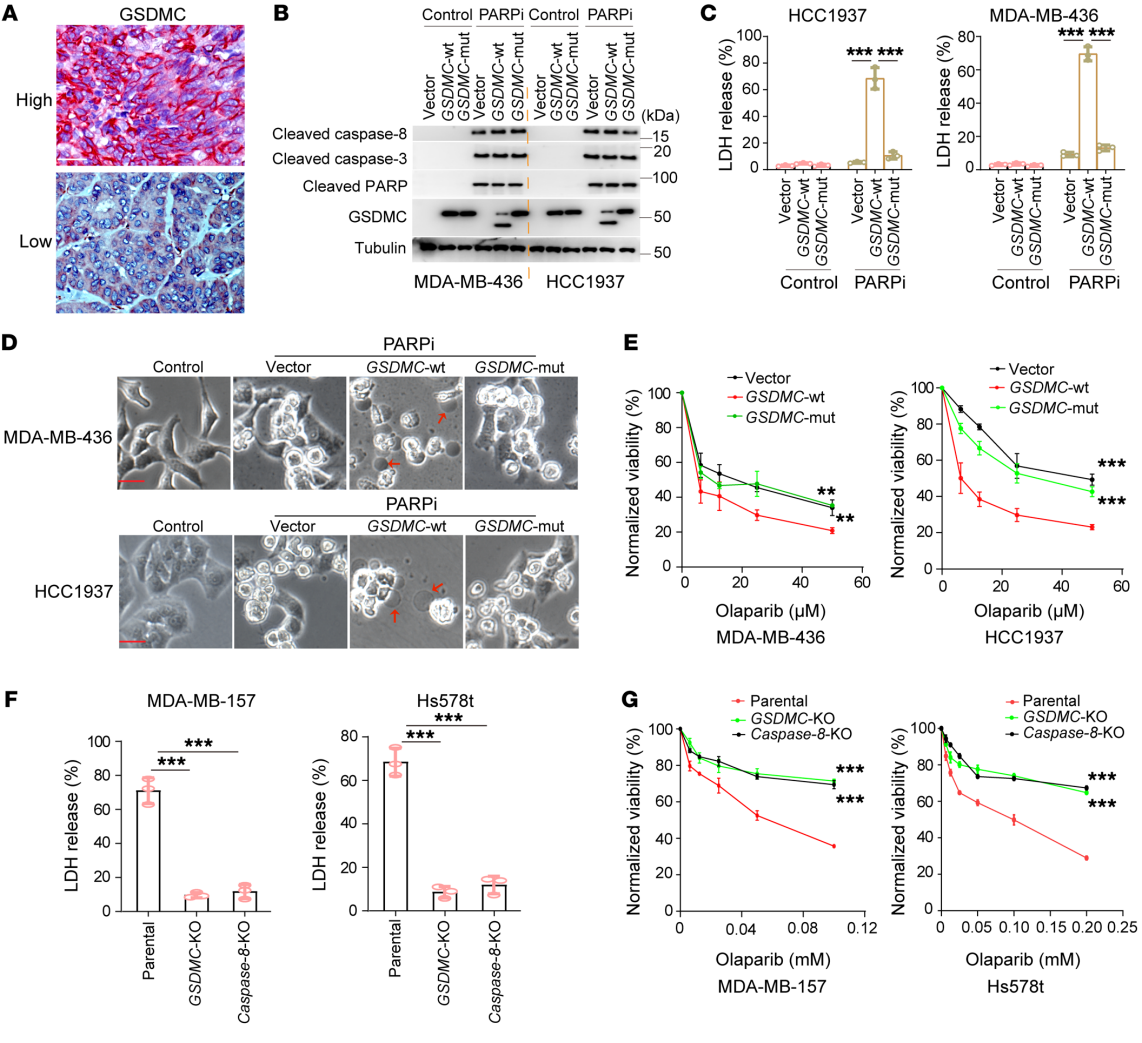
GSDMC-mediated CCP enhances the cytotoxicity of PARPi. (**A**) Representative IHC staining results for GSDMC in human TNBC tissues. Scale bar: 50 μm**.** (**B**) MDA-MB-436 and HCC1937 cells harboring an empty vector (vector) or expressing WT *GSDMC* (*GSDMC*-WT) or the D365A mutant (*GSDMC*-mut). Immunoblotting demonstrating caspase-8 cleavage of GSDMC in indicated cells treated with olaparib (20μM) for 48 hours. (**C**) Cells in **B** were treated with olaparib (20 μM) for 72 hours. Cell death measured by LDH release (LDH-released cell death) is shown (*n* = 3). (**D**) Same treatment as in **C**; representative images of dying cell morphology. Red arrows indicate cell swelling with large bubbles. Scale bar: 20 μm. (**E**) Cells in **B** were treated with the indicated concentrations of olaparib for 72 hours and subjected to a cell viability assay (*n* = 3). (**F**) MDA-MB-157 and Hs578t cells with deletion of *GSDMC* or caspase-8 were treated with olaparib (100 μM) for 72 hours. LDH-released cell death is shown (*n* = 3). (**G**) Cells in **F** were treated with the indicated concentrations of olaparib for 72 hours and subjected to a cell viability assay (*n* = 3). Data represent mean ± SD. 1-way ANOVA was used. **P < 0.01, ***P < 0.001.

To address potential relationship between GSDMC and response to PARPi, we first analyzed the expression level of GSDMC in TNBC and its clinical correlation. The results showed that 38.5% of patients with TNBC exhibited high GSDMC expression in tumor tissue (Figure 1A, Table 1, and Supplemental Figure 1; supplemental material available online with this article; https://doi.org/10.1172/JCI166841DS1), which, interestingly, showed better response to PARPi therapy (Table 2). Next, we asked whether GSDMC-mediated CCP might affect tumor response to PARPi. To this end, we stably expressed WT and caspase-8 cleavage site–mutant *GSDMC* in BRCA-mutant MDA-MB-436 and HCC1937 cells (Supplemental Figure 2, A–C). PARPi induced caspase-8 cleavage of GSDMC (Figure 1B) and pyroptotic cell death (Figure 1, C and D), as evidenced by LDH release and bubble-like protrusions induced by WT *GSDMC*. While *GSDMC* mutation and vector control did not show PARPi-induced CCP, instead, typical apoptosis characteristics were observed (Figure 1, C and D), indicating that GSDMC expression switched apoptosis to pyroptosis, as shown previously (8) in response to PARPi treatment. In addition, WT *GSDMC* increased the cytotoxicity of PARPi (Figure 1E). We knocked out *GSDMC* or caspase-8 in GSDMC-positive MDA-MB-157 and Hs578t (Supplemental Figure 2, D and E), which blocked PARPi-induced CCP (Figure 1F). Deletion of *GSDMC* or caspase-8 significantly decreased sensitivity of tumor cells to PARPi (Figure 1G). Thus, these data suggest that GSDMC-mediated CCP may sensitize tumor cells to PARPi in vitro.

**Figure 2:**
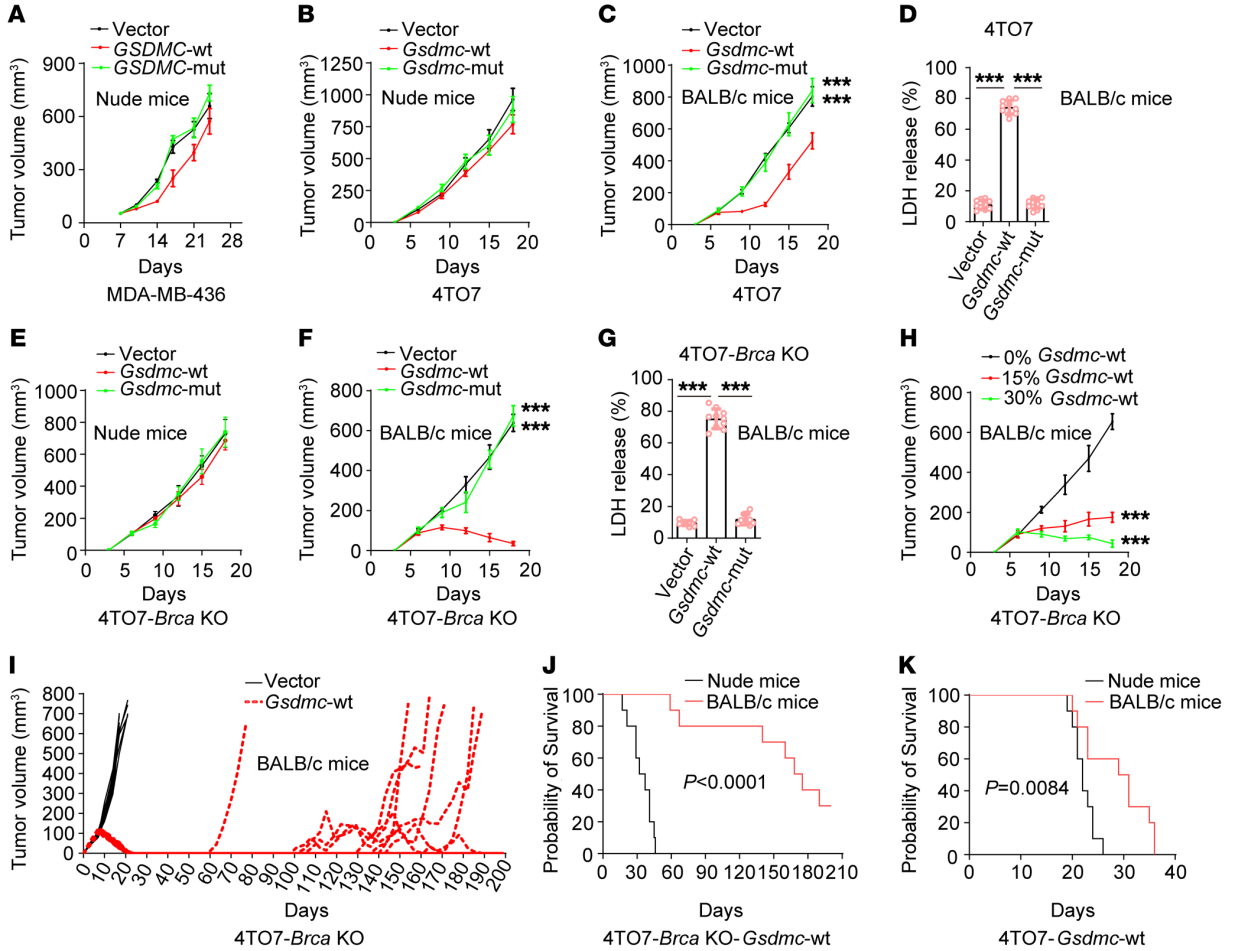
GSDMC-increased sensitivity of PARPi is immune-mediated in vivo. (**A**) MDA-MB-436 stable cells as indicated were inoculated into the mammary fat pad of nude mice (*n* = 10). Mice were administered olaparib. Tumor growth was shown. (**B** and **C**) 4TO7 cells harboring an empty vector (vector) or expressing WT mouse *Gsdmc* (*Gsdmc*-WT) or the caspase-8 cleavage site D263A mutant (*Gsdmc*-mut) were inoculated into the mammary fat pad of nude mice (**B**) or immunocompetent BALB/c mice (**C**) (*n* = 10). Mice were administered olaparib. Tumor growth was shown. (**D**) LDH level in tumor slurry of tumors indicated in **C** was measured. (**E** and **F**) Same as **B** and **C**, except that stable transfectants were established in 4TO7-*Brca* KO instead of 4TO7 parental cells and injected (*n* = 10). (**G**) LDH level in slurry of tumors indicated in **F** was measured. (**H**) Parental 4TO7 cells mixed with 0%, 15%, or 30% 4TO7-*Brca*–KO *Gsdmc*-WT cells were inoculated into BALB/c mice (*n* = 10). Mice were administered olaparib. Tumor growth was shown. (**I**) BALB/c mice with 4TO7-*Brca*–KO *Gsdmc*-WT or vector tumors were administered olaparib and durable tumor regression was monitored (*n* = 10). (**J** and **K**) 4TO7-*Gsdmc*-WT (**J**) or 4TO7-*Brca*–KO *Gsdmc*-WT (**K**) cells were inoculated into the mammary fat pad of nude mice and immunocompetent BALB/c mice (*n* = 10). Mice were administered olaparib. Survival curves were shown. Data represent mean ± SD. 1-way ANOVA was used for **A**–**H**. The log-rank test was used for **J** and **K**. ***P < 0.001.

However, in nude mice models, different results were observed. Increased sensitivity of PARPi by GSDMC was neither observed in human cell line MDA-MB-436 xenograft tumor model (Figure 2A), nor in mouse cell line, 4TO7 (Figure 2B) that was ectopically expressed *Gsdmc*-WT or *Gsdmc*-mut (Supplemental Figure 3A). Interestingly, in immunocompetent mice model, *Gsdmc*-WT expression suppressed tumor growth compared with *Gsdmc*-mut or vector treated with PARPi (Figure 2C), with elevated CCP in 4TO7-*Gsdmc*-WT tumors (Figure 2D). Similar to 4TO7 cells, *Gsdmc*-WT expression in 4TO7-*Brca*–KO cells did not slow down PARPi-treated tumor growth compared with *Gsdmc*-mut or vector in nude mice (Figure 2E and Supplemental Figure 3, B and C), but promoted superior tumor clearance in immunocompetent mice (Figure 2F), with enhanced CCP (Figure 2G). We mixed the parental 4TO7 cells with stable 4TO7-*Brca*–KO *Gsdmc*-WT cells together in different ratios. Notably, 15% or 30% of 4TO7-*Brca*–KO cells with *Gsdmc*-WT expression were sufficient to reduce tumor growth in response to PARPi treatment (Figure 2H), indicating GSDMC-mediated potent antitumor immunity. *Gsdmc*-WT expression led to long-term rejection of 4TO7-*Brca*–KO tumors in immunocompetent mice treated with PARPi, with tumors taking an average of 120.5 days to regrow to 100 mm^3^ after tumor clearance (Figure 2I), indicating sustained antitumor immunity. Importantly, *Gsdmc*-WT expression extended the survival of immunocompetent mice bearing 4TO7 (Figure 2J) or 4TO7- *Brca*–KO tumors (Figure 2K) compared with that of nude mice under PARPi treatment. Taken together, these data suggest that antitumor immunity is required for GSDMC-enhanced efficacy of PARPi in vivo.

### GSDMC-mediated CCP suppressed tumor growth by increasing tumor infiltration and activation of cytotoxic and memory T cells.

**Figure 3:**
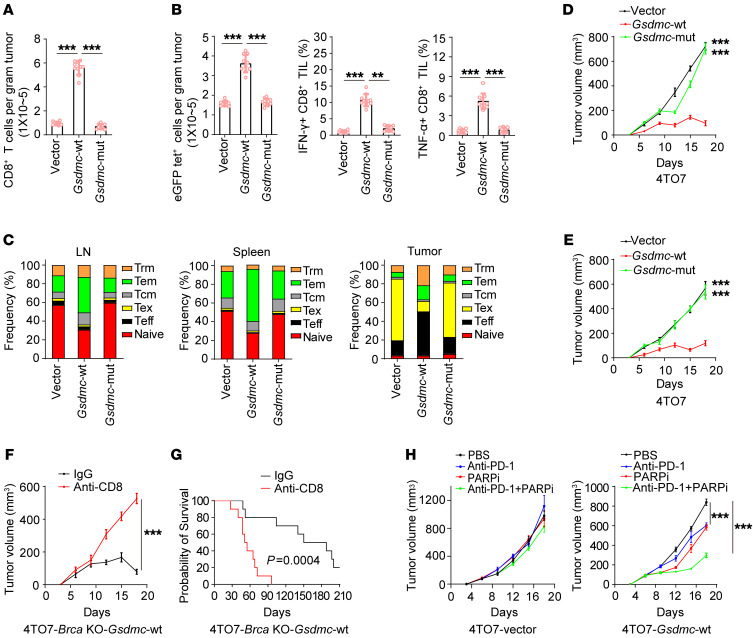
GSDMC-mediated CCP increases the population and tumor infiltration of memory T cell. (**A**) 4TO7-*Brca*–KO cells stably expressing an empty vector (vector) or WT mouse *Gsdmc* (*Gsdmc*-WT) or the D263A mutant (*Gsdmc*-mut) were inoculated into the mammary fat pad of immunocompetent BALB/c mice (*n* = 10). Mice were administered olaparib. Percentage of tumor-infiltrating CD8^+^ T cells was analyzed. (**B**) Overexpression of *eGFP* in the stable cells as indicated in **A**. Then cells and mice were treated same as in **A**. Mean numbers of eGFP tetramer^+^ (eGFP tet^+^) CD8^+^ T cells per gram of tumor (left). Percentage of IFN-γ^+^ (middle) or TNF-α^+^ (right) CD8^+^ T cells activated by eGFP peptide. (**C**) Frequency of memory T cell subsets in lymph node (LN), spleen, and tumors of **A.** Tex, exhausted T cell. (**D**) Initial tumor challenge and mice treatment were same as in **A**. Tumors were removed on day 18. Then tumor rechallenge of 4TO7 parental cells was performed 60 days after tumor removal. Tumor growth was shown (*n* = 10). (**E**) Stable cells as indicated in **A** were inoculated into the mammary fat pad of immunocompetent BALB/c mice (*n* = 10). 4TO7 parental cells were simultaneously injected into contralateral mammary fat pad. Mice were administered olaparib. Tumor growth of 4TO7 parental cells was monitored. (**F** and **G**) 4TO7-*Brca*–KO *Gsdmc*-WT cells were inoculated into BALB/c mice (*n* = 10). Mice were administered olaparib. Depletion of CD8^+^ T cell with anti-CD8. Curves of tumor growth (**F**) and survival (**G**). (**H**) Growth curve of 4TO7-*Gsdmc*-WT and 4TO7-vector tumors in BALB/c mice (*n* = 10) treated with olaparib or PD-1 antibody or the combination. Data represent mean ± SD. 1-way ANOVA was used for **A**, **B**, **D**, **E**, and **H**. Unpaired 2-tailed *t* test was used for **F**. Log-rank test was used for **G**. **P < 0.01, ***P < 0.001.

To investigate the mechanism of GSDMC-mediated antitumor immunity, we analyzed the tumor-infiltrating immune cells under PARPi treatment. *Gsdmc*-WT expression decreased the tumor infiltration of Tregs (Supplemental Figure 4A), while increasing that of activated DC and CD4^+^ T cells (Supplemental Figure 4, B and C), compared with *Gsdmc*-mut or vector in 4TO7-*Brca*–KO tumors. For NK cells and tumor-specific TAM, no significant difference was observed (Supplemental Figure 4, D and E). Importantly, *Gsdmc*-WT expression significantly elevated tumor-infiltrating CD8^+^ T cell population (Figure 3A and Supplemental Figure 5A). More functional CD8^+^ T cells that were tumor-specific (eGFP tet^+^) or expressed IFN-γ or TNF-α were observed in *Gsdmc*-WT tumors (Figure 3B and Supplemental Figure 5B). The sustained tumor growth inhibition in Figure 2I suggested the protection of memory CD8^+^ T cells against tumors. Indeed, *Gsdmc*-WT increased memory CD8^+^ T cells in mice with 4TO7-*Brca*–KO tumors compared with *Gsdmc*-mut or vector, with increased effector memory T cells (Tems) and central memory T cells (Tcms) in lymph nodes, increased Tems in spleen, increased Tems and tissue-resident memory T cells (Trms) in tumors, which resulted in marked upregulation of effector T cells (Teffs) in tumors (Figure 3C). The protection role of memory CD8^+^ T cells induced by *Gsdmc*-mediated CCP in response to PARPi treatment was further confirmed by reduced tumor growth of 4TO7 parental cells in rechallenged (Figure 3D) and bilateral (Figure 3E) tumor models. Consistently, depletion of CD8^+^ T cells with antibody reversed the tumor growth inhibition (Figure 3F) and shortened overall survival (Figure 3G) in mice with 4TO7-*Brca*–KO tumors expressing *Gsdmc*-WT. Notably, combination of PARPi and anti-PD-1 caused a marked shrinkage in volume of 4TO7 tumors expressing *Gsdmc*-WT (Figure 3H), indicating the contribution of GSDMC to the combination therapy. Thus, during the PARPi treatment GSDMC-mediated CCP augments antitumor immunity by promoting the activation and tumor infiltration of cytotoxic and memory T cells.

### T cell–released granzyme B induced CCP by caspase-6 cleavage of GSDMC.

**Figure 4:**
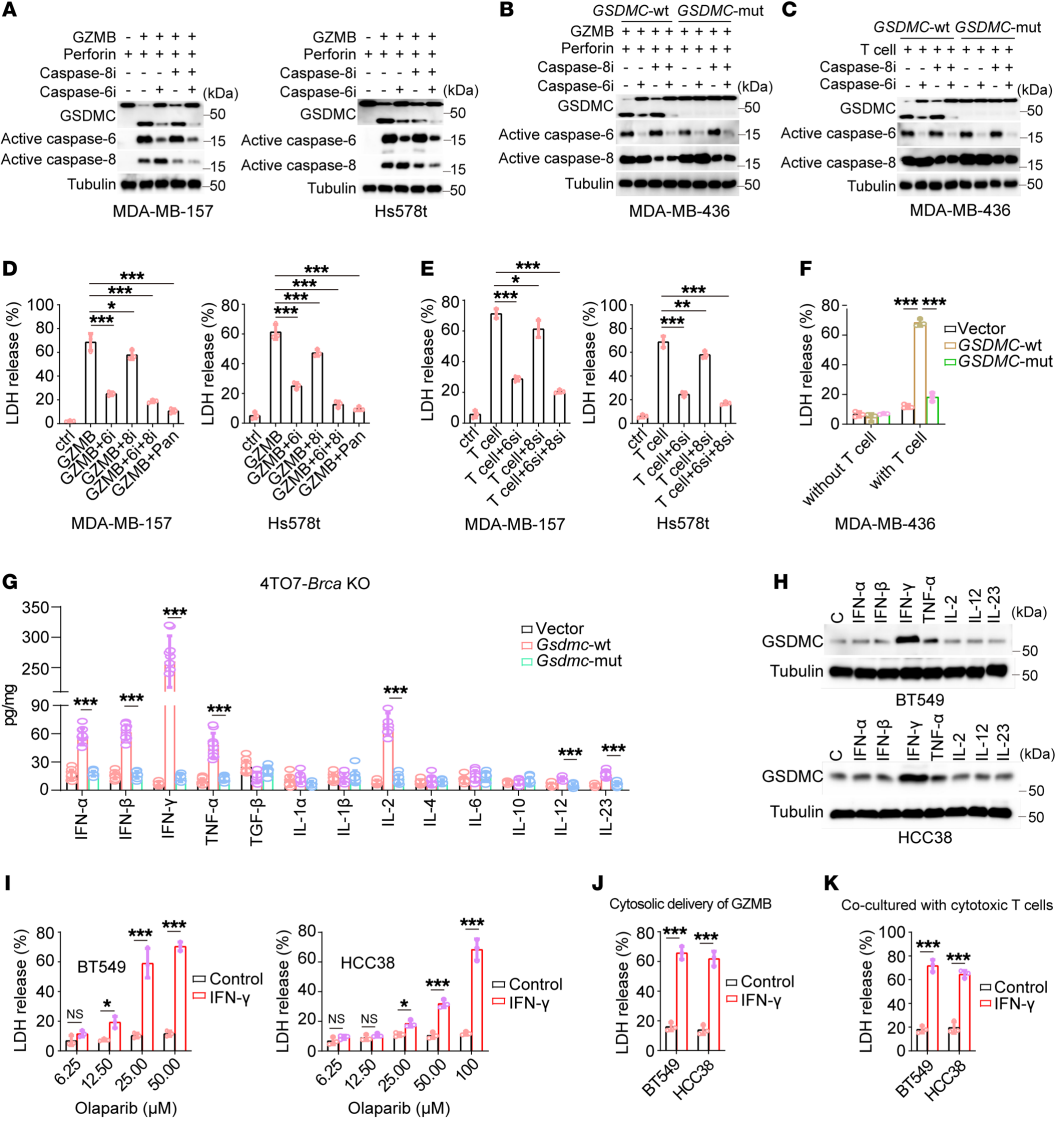
T cell–derived GZMB induces CCP by caspase-6 cleavage of GSDMC, and IFN-γ promotes GSDMC expression to enhance the killing effect of T cells and PARPi. (**A**) GZMB-mediated GSDMC cleavage by caspase-6 in MDA-MB-157 and Hs578t cells. Caspase-6i, caspase-6 inhibitor; caspase-8i, caspase-8 inhibitor. (**B**) MDA-MB-436 cells expressing WT *GSDMC* (*GSDMC*-WT) or the D365A mutant (*GSDMC*-mut) were treated with GZMB or inhibitors of caspase-6 or caspase-8. Immunoblotting of GSDMC cleavage. (**C**) Same as **B**, except that cells were cocultured with T cells instead of GZMB treatment. (**D**) Cell death measured by LDH release (LDH-released cell death) induced by GZMB in MDA-MB-157 and Hs578t cells (*n* = 3). (**E**) LDH-released cell death induced by cytotoxic T cells in MDA-MB-157 and Hs578t cells treated with caspase-6 siRNA (6si) and/or caspase-8 siRNA (8si) (*n* = 3). (**F**) LDH-released cell death induced by cytotoxic T cell in MDA-MB-436 cells with expression of vector, *GSDMC*-WT, and *GSDMC*-mut (*n* = 3). (**G**) Quantification of cytokine levels by ELISA in tumors of Figure 2F. (**H**) GSDMC induction by cytokines indicated in BT549 and HCC38 cells. (**I**) IFN-γ enhanced LDH-released cell death induced by olaparib at indicated concentration in BT549 and HCC38 cells (*n* = 3). (**J** and **K**) IFN-γ enhanced LDH-released cell death in BT549 and HCC38 cells treated with cytosolic delivery of GZMB (**J**) or cocultured with cytotoxic T cells (**K**) (*n* = 3). Data represent mean ± SD. 1-way ANOVA was used for **D**–**F**. 2-way ANOVA was used for **I**. Unpaired 2-tailed *t* test was used for **G**, **J**, and **K**. *P < 0.05, **P < 0.01, ***P < 0.001.

T cell–derived granzyme B (GZMB) activates caspase-6 in target cells (15). Our previous study showed that GSDMC can be cleaved by caspase-6 (8) in addition to caspase-8. We therefore asked whether GZMB might trigger CCP though caspase-6 cleavage of GSDMC. Cytosolic delivery of GZMB with perforin induced GSDMC cleavage that could be markedly diminished by caspase-6 inhibitor in MDA-MB-157 and Hs578t cells (Figure 4A). Considering the similar cleavage patterns of GSDMC between caspase-6 and caspase-8 (8), we speculated that caspase-6 shares the same cleavage site as that of caspase-8. Indeed, mutation of caspase-8 cleavage site D365 to A in GSDMC abolished the caspase-6–mediated GSDMC cleavage in MDA-MB-436 cells treated with GZMB delivery (Figure 4B) or cocultured with T cells (Figure 4C). Importantly, cytosolic delivery of GZMB resulted in extensive CCP that was then suppressed by caspase-6 inhibitor in MDA-MB-157 and Hs578t cells (Figure 4D). Consistently, coculture of cytotoxic T cells with MDA-MB-157 or Hs578t cells also caused extensive CCP that could be suppressed by caspase-6 siRNA (Figure 4E and Supplemental Figure 6). Further, ectopic expression of GSDMC-WT but not D365A mutant mediated CCP in MDA-MB-436 cells cocultured with cytotoxic T cells (Figure 4F). Taken together, these data suggest that T cell–derived GZMB induced CCP mainly via caspase-6 cleavage of GSDMC.

### IFN-γ elevated GSDMC expression and thereby exacerbated PARPi- and T cell–mediated CCP.

Given that gasdermin proteins could be induced by cytokines (12), we detected the expression level of immunostimulatory cytokines in a tumor slurry. IFN-α, IFN-β, IFN-γ, TNF-α, IL-2, IL-12, and IL-23 were increased in 4TO7-*Brca*–KO tumors expressing *Gsdmc*-wt compared with *Gsdmc*-mut or vector (Figure 4G), of which only IFN-γ evidently upregulated GSDMC expression in BT549 and HCC38 cells (Figure 4H). Notably, IFN-γ enhanced PARPi-mediated CCP in BRCA-proficient BT549 and HCC38 cells (Figure 4I). Similarly, IFN-γ–enhanced CCP was also observed in BT549 and HCC38 cells treated with GZMB delivery (Figure 4J) or cocultured with cytotoxic T cells (Figure 4K). These data indicate that GSDMC-mediated CCP kills tumor cells and boosts antitumor immunity in an exacerbating feedback manner in response to PARPi treatment.

### GSDMC-fueled PARPi efficacy in both BRCA-proficient and -deficient tumors in multiple cancer types, but BRCA deficiency reduced more tumor growth in GSDMC-positive tumors.

**Figure 5:**
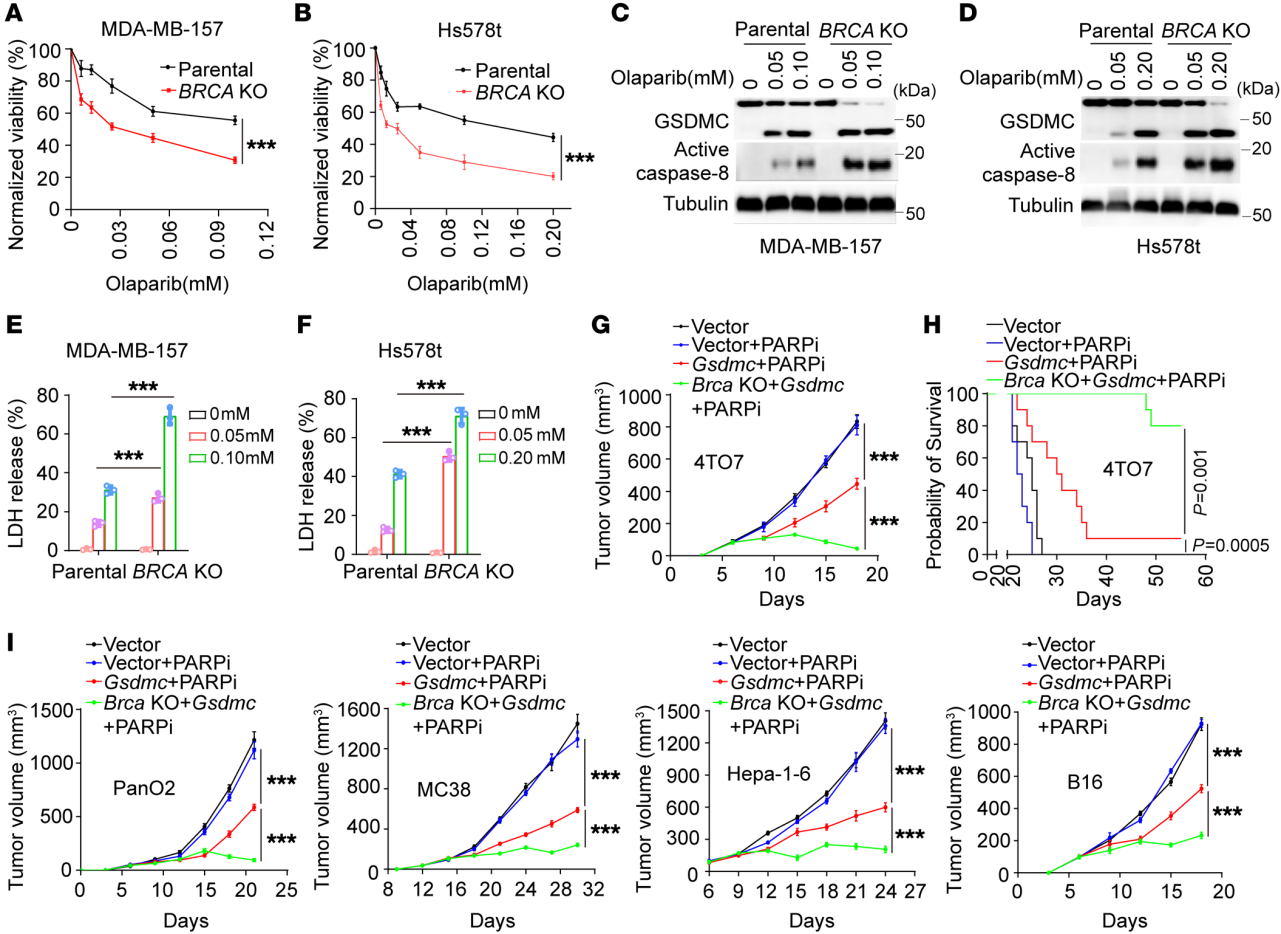
GSDMC contributes to PARPi efficacy in both BRCA-proficient and -deficient tumors. (**A**–**F**) MDA-MB-157 and Hs578t cells with deletion of *BRCA*. Cells were treated with the indicated concentrations of olaparib for 72 hours and subjected to a cell viability assay (*n* = 3) (**A** and **B**). Immunoblotting of GSDMC cleavage in cells treated with the indicated concentrations of olaparib for 72 hours (**C** and **D**). Cell death measured by LDH release (LDH-released cell death) induced by olaparib at the indicated concentrations (*n* = 3) (**E** and **F**). (**G** and **H**) 4TO7 parental or *Brca*-KO cells with ectopic expression of *Gsdmc* were inoculated into the mammary fat pad of immunocompetent BALB/c mice (*n* = 10). Mice were administered olaparib (50 mg/kg) 5 times per week for 18 days. Tumor growth (**G**) and survival (**H**) curves were shown. (**I**) Ectopic expression of *Gsdmc* in parental or *Brca*-KO cells of PanO2, MC38, Hepa-1-6, B16. Cells were inoculated into the mammary fat pad of immunocompetent C57BL/6 mice (*n* = 10). Mice were treated same as **G**. Tumor growth curves were shown. Data represent mean ± SD. Unpaired 2-tailed *t* test was used for **A** and **B**. 2-way ANOVA was used for **E** and **F**. 1-way ANOVA was used for **G** and **I**. Log-rank test was used for **H**. ***P < 0.001.

GSDMC-increased efficacy of PARPi was observed in BRCA-deficient (Figure 1E and Figure 2F) and BRCA-proficient (Figure 1G and Figure 2C) cells, indicating that BRCA deficiency is not absolutely required for, but seems to strengthen, the effects for GSDMC-sensitizing tumor cells to PARPi. To further investigate the influence of BRCA on PARPi efficacy in GSDMC-positive tumors, we deleted *BRCA* in MDA-MB-157 and Hs578t cells (Supplemental Figure 7, A and B). Despite the GSDMC-mediated CCP promoted PARPi killing effect in BRCA-proficient MDA-MB-157 and Hs578t (Figure 1G), *BRCA* deletion significantly reduced cell viability compared with parental cells in responding to PARPi treatment (Figure 5, A and B). Moreover, *BRCA* deletion facilitated caspase-8 cleavage of GSDMC (Figure 5, C and D) and thus caused extensive CCP (Figure 5, E and F) compared with parental cells in MDA-MB-157 and Hs578t treated with PARPi. *Gsdmc*-WT expression induced increased PARPi sensitivity in *Brca*-deficient tumors compared with *Brca*-proficient ones in BALB/c mice (Figure 2, C and F), suggesting that *Brca* deficiency may contribute to *Gsdmc*-WT–increased PARPi sensitivity in vivo. Thus, we stably expressed *Gsdmc* in 4TO7 and 4TO7-*Brca*–KO cells (Supplemental Figure 7C). Compared with the vector, *Gsdmc* significantly suppressed 4TO7 tumor growth and extended overall survival in mice treated with PARPi, while nearly complete tumor rejection and the longest survival were observed in 4TO7-*Brca*–KO tumors expressing *Gsdmc* (Figure 5, G and H). To ascertain the universality of GSDMC-enhanced PARPi sensitivity in cancers, we stably enforced *Gsdmc* expression in parental and *Brca*-KO murine cancer cells, including PanO2 pancreatic cancer, MC38 colorectal cancer, Hepa-1-6 liver cancer, and B16 melanoma cells (Supplemental Figure 7, D and E). Similar to murine 4TO7 breast cancer, *Gsdmc* sensitized tumor cells to PARPi in BRCA-proficient parental cells, whereas greater tumor inhibition was shown in *BRCA*-KO cancer cells with *Gsdmc* expression in PanO2, MC38, Hepa-1-6, and B16 cells (Figure 5I). Taken together, these data suggest that GSDMC increases PARPi sensitivity in both BRCA-proficient and -deficient tumors with much more potency in the BRCA-deficient cancer cells.

## Discussion

Here, we show that GSDMC expression increases PARPi sensitivity through CCP-induced memory T cell expansion in the tumor microenvironment and in lymphoid organs, which enhances antitumor immunity in multiple cancer types. Protection by vaccination is the gold standard for ICD (16). PARPi-induced CCP of GSDMC-positive 4TO7 protected mice from rechallenge with parental 4TO7, indicating that GSDMC-mediated CCP generates potent immunity against tumors via ICD. PARPi directly triggers CCP through GSDMC cleavage by caspase-8, or indirectly by caspase-6, which is activated by GZMB. PARPi-induced CCP augmented memory T cell expansion both in tumor microenvironment and lymphoid organs, which boosted potent T cell response. IFN-γ derived from CCP-induced immune response upregulates GSDMC expression, which augments antitumor immunity and PARPi sensitivity in an exacerbating feedback manner (Supplemental Figure 8). In spite of caspase-8 activation, caspase-6 activation was not observed in MDA-MB-157 and Hs578t cells treated with PARPi in vitro (data not shown), indicating that caspase-6 was not involved in PARPi-induced caspase cascade that caused cell death in these cell types. Both caspase-6 and -8 are activated by intracellular delivery of GZMB (Figure 4A). Caspase-8 is involved in the process of GZMB cleavage of GSDMC, but caspase-6 plays a dominant role in the process (Figure 4A).

According to the identified mechanisms in the study, the sensitization effect of GSDMC in tumor suppression may not be limited to PARPi. Theoretically, GSDMC can sensitize tumor cells to the treatment of any drugs that could activate caspase-6 or caspase-8, or could stimulate antitumor immunity response. Thus, GSDMC-mediated CCP may have a broader influence on multiple drugs including chemotherapy and radiation.

Increased TNF-α was observed in PARPi-treated tumor slurry. We previously showed that TNF-α induces CCP through caspase-8 cleavage of GSDMC (8). Thus, we speculate that TNF-α may also contribute to the PARPi killing effect in GSDMC-positive cancer cells. As a form of “dirty death”, pyroptotic cell–released cellular contents stimulate immune response (17). The critical cellular component that activates and sustains antitumor immunity should be further determined in future. Besides GSDMC, other gasdermin proteins have been shown to have pyroptotic capability or potential (12, 18, 19). Thus, PARPi-induced CCP may occur in tumor cells with a gasdermin expression in addition to GSDMC.

We previously reported that PD-L1 blockade sensitizes PARPi-treated tumor cells to T cell killing (20). Combined PARPi and immune checkpoint therapy is ongoing in clinical trials (21). Revealed clinical data showed modest clinical activity of the combination (22). False negative PD-L1 staining by heavy glycosylation may lead to inaccurate prediction of clinical outcome (23, 24). Based on this study, gasdermin-mediated CCP may also serve as a marker to stratify patients for maximum benefits of combination therapy.

In contrast to the significant suppression of tumor growth in vitro (Figure 1, E and G), GSDMC-mediated CCP slightly suppressed tumor growth in response to PARPi treatment in nude mice (Figure 2, A and B). It is conceivable that the tumor microenvironment causes the different antitumor effect of PARPi between in vitro experiments and in nude mice. However, GSDMC expression mediated CCP while vector mediated apoptosis (Figure 1C), which results in different outcomes in immunocompetent mice (Figure 2C). Our data showed that it is the cell death pattern, but not the extent of cell death, that plays the critical role in tumor suppression. PARPi triggers pyroptosis, not apoptosis, which is a “clean death” that inhibits immune response, in GSDMC-positive tumor cells, providing a new perspective for PARPi treatment that cell death pattern is another important consideration beyond BRCA mutation. Sufficient expression and distinct protease cleavage of a gasdermin in tumor cells are required for CCP (17). Therefore, gasdermin expression and activation of the protease that cleaves the gasdermin under PARPi treatment is the prerequisite for CCP occurrence. Clinical or preclinical testing of PARPi-induced CCP could be extended in additional cancer types beyond those in this study.

In both BRCA-deficient and -proficient tumors, GSDMC enhances PARPi sensitivity. Nevertheless, PARPi works better in BRCA-deficient tumors than in BRCA-proficient tumors, indicating that BRCA mutation is still a good marker for PARPi treatment even in GSDMC-positive cancer types. This may be attributed to the mechanism that apoptosis and pyroptosis share the same upstream pathway, and BRCA deficiency enhances PARPi-triggered apoptotic events, such as caspase activation, that cleaves GSDMC. GSDMC partially overcame PARPi resistance in cells with BRCA reversion mutation (Supplemental Figure 9, A and B), but not in cells with PARP1 loss (Supplemental Figure 9, C and D). It is conceivable that loss of PARP1 causes olaparib to lose its target and become ineffective in activating apoptotic caspases, thus, GSDMC may not be effectively activated. CCP of 15% of tumor cells is sufficient to inhibit tumor growth in response to PARPi treatment, suggesting that CCP-augmented antitumor immunity, but not direct killing of PARPi, plays a central role in PARPi-induced tumor regression. This study emphasizes the potential value of GSDMC as a general biomarker that expands the population of patients with multiple cancer types likely to benefit from PARPi beyond BRCA mutation carriers.

It has been reported that high mobility group box 1 (HMGB1) could both promote tumor cell proliferation (25) and enhance T cell–dependent antitumor immunity (26). We observed that HMGB1 indeed promoted proliferation of MDA-MB-436 and 4TO7 cells (Supplemental Figure 10, A and B). However, HMGB1 was not increased in *GSDMC*-WT cells compared to vector control and *GSDMC*-mut under PARPi treatment in vitro (Supplemental Figure 10, C and D). In contrast, a massive release of HMGB1 in *GSDMC*-WT tumors was evidenced in nude mice and immunocompetent BALB/c mice (Supplemental Figure 10, E and F). Given the lack of T cells in nude mice, the GSDMC-increased sensitivity of tumor cells to PARPi in vitro may be offset by HMGB1-fueled tumor cell proliferation in nude mice, while in immunocompetent BALB/c mice, HMGB1-enhanced cytotoxicity of T cells may overcome the tumor proliferation-promoting effect of HMGB1, suggesting the dominant role of T cell–mediated antitumor immunity in response to PARPi treatment in normal immunocompetent mice. These data explain the reasons for the inconsistency of in vivo nude mice and in vitro cell culture experimental data.

Genomic instability is one of the critical hallmarks of cancer (27). DNA damage repair (DDR) defects in cancer cells create vulnerabilities, which could potentially be exploited to develop anticancer drugs. Efforts to probe potential DDR targets for anticancer treatment have produced numerous inhibitors that inhibit other key DDR components than PARP, including ATR, ATM, WEE1, DNA-PK, CHK1, and CHK2 (4), and some of them have been in early stage clinical trials. Recent studies reported that receptor tyrosine kinases (RTKs) such as c-Met and ALK contributed to PARPi resistance, and combined inhibition of these RTKs with PARPi induced synthetic lethality in breast cancer, liver cancer, pancreatic cancer, and ovarian cancer (28–33). The study raises a possibility that GSDMC may sensitize tumor cells to these DDR inhibitors as it does to PARPi. Thus, assessment of GSDMC expression level in tumor tissues may help optimize clinical trials of these DDR inhibitors.

## Methods

### Cell culture.

MDA-MB-436, HCC1937, MDA-MB-157, Hs578t, BT549, HCC38, 4TO7, PanO2, MC38, Hepa-1-6, and B16 cells were obtained from ATCC and cultured in DMEM or RPMI-1640 medium with 10% FBS. All cell lines were verified to be free of mycoplasma by PCR and validated by short tandem repeat DNA fingerprinting.

### Plasmids, antibodies, and reagents.

Human *GSDMC* gene and its D365A mutant, caspase-8–KO, and GSDMC-KO constructs were generated as previously reported (8). Mouse *Gsdmc* (EX-Mm12297-M39) was purchased from GeneCopoeia and was cloned to the lentiviral vector pCDH-CMV for overexpression. Q5 site-directed mutagenesis kit (E0554S, New England BioLabs) was used to generate the D263A mutant of *Gsdmc*. Human (sc-400093) and mouse (sc-419362) CRISPR/Cas9 systems for BRCA KO were purchased from Santa Cruz Biotechnology. All plasmids were verified by sequencing.

The following antibodies were used for immunoblotting at 1:1,000: α-GSDMC (GTX33979, GeneTex), α-GSDMC (27630-1-AP, Proteintech), α-cleaved caspase-8 (NB100-56116, Novus Biologicals), α-cleaved caspase-6 (9761, Cell Signaling Technology), α-cleaved caspase-3 (NB100-56113, Novus Biologicals), α-cleaved PARP (NB100-56599, Novus Biologicals), α-caspase-8 (4790, Cell Signaling Technology), α-BRCA (NBP1-41185, Novus Biologicals), α-Tubulin (NB100-690, Novus Biologicals), α-Vinculin (4650, Cell Signaling Technology), and α-Flag (F1804, Sigma Aldrich).

Olaparib (S1060) was purchased from Selleck Chemicals. siRNA for caspase-6 (sc-72802) and caspase-8 (sc-29930) were purchased from Santa Cruz Biotechnology. GZMB (LS-G134962-5) and perforin (LS-G26593-10) proteins were from LifeSpan BioSciences. IFN-α (rcyc-hifna1), IFN-γ (rcyec-hifng), and IL-12 (rcyc-hil12) were purchased from InvivoGen. IFN-β (Z03109) was purchased from GenScript. TNF-α (ab9642) was purchased from Abcam. IL-2 (HZ-1015) was purchased from Proteintech Group. IL-23 (PHC9321) was purchased from Thermo Fisher Scientific. Pan caspase inhibitor (Z-VAD-FMK; FMK001), caspase-6 (Z-VEID-FMK; FMK006), and caspase-8 (Z-IETD-FMK; FMK007) inhibitors were purchased from R&D Systems.

### Generation of stable transfectants.

Gene KO stable cells were generated as described previously (8). Lentiviral-based plasmids containing sgRNAs targeting human *GSDMC* (GGTAACAATTTGAAACTCGA), human *caspase-8* (GCCTGGACTACATTCCGCAA), human *BRCA* (TGGATTTCGCAGGTCCTCAA), and mouse *BRCA* (GTACCCAAAGTCTCGTCAAG) were purchased from GenScript. Lentiviruses were produced and all stable cells were generated as previously reported (8). Single cell clones were isolated, cultured, and validated by immunoblotting. Successful KO clones were pooled to establish stable cell lines. For stable cells expressing vector, *GSDMC*-WT, *GSDMC*-mut, *Gsdmc*-WT, and *Gsdmc*-mut, all genes were cloned to the lentiviral-based plasmids. For eGFP-labeled stable cells, lentiviral pGIPZ-*eGFP* empty vector was used.

### LDH release assay.

Detection of released LDH was performed as previously described (8).

### Patients with TNBC and IHC staining.

Tumor samples from 200 patients with TNBC for evaluation of GSDMC expression level were obtained from Xiangya Hospital, Central South University. Tumor samples from 60 patients with TNBC treated with PARPi for analysis of correlation between GSDMC expression level and PARPi response rate were obtained from Xiangya Hospital and Biotech Company (Xi’an, China). IHC staining was performed as previously reported (8). Briefly, tissue samples were incubated with the GSDMC antibody (GTX33979, GeneTex) and then the secondary antibody conjugated with biotin (P0615, Beyotime Biotechnology), and, finally, incubated with an avidin-biotin-peroxidase complex. According to histologic scoring, the IHC staining intensity was ranked into 1 of 2 groups: high (score 3 or score 2) and low (score 1 or score 0). According to the RECIST (Response Evaluation Criteria in Solid Tumors), therapeutic effects of PARPi were evaluated. Complete response and partial response were classified as PARPi-sensitive, while stable disease and progressive disease were classified as PARPi-resistant.

### Cell viability assay.

Cell viability was analyzed as reported previously (29). Briefly, 1,500 cells were seeded in a 96-well plate and treated with olaparib at indicated concentrations for 72 hours. Fresh medium with 100 μM resazurin was then added into cells. After 1 hour, cell viability was analyzed at spectra of 560EX nm/590EM nm. Survival curves were shown as mean ± SD compared with DMSO.

### Immunoblotting.

Immunoblotting analyses were performed as reported previously (8).

### FACs analysis of lymphocytes in LN, spleen, and tumor.

LN, spleen, and tumors were harvested and minced into small pieces using sterile scissors, followed by digestion in RPMI-1640 with 2% FBS, 2 μg/μl collagenase (11088866001, Sigma-Aldrich) and 100 μg/mL DNase I (11284932001, Sigma-Aldrich). Cell clumps were filtered with 70 μm strainers. Cell suspension was centrifuged and the obtained cell pellet was washed with PBS. Lymphocytes were isolated by Percoll-gradient centrifugation and washed with Leibovitz’s L-15 medium (11415064, Thermo Fisher Scientific). Cells were stained with following antibodies: CD45RA (clone 14.8) from BD Bioscience; PD-1 (clone J43) from Thermo Fisher Scientific; and CD45RO (clone UCHL1), CD3 (clone 17A2), CD8a (clone 53–6.7), CD4 (clone GK 1.5), FOXP3 (clone MF-14), CD49b (clone DX5), NKp46 (clone 29A1.4), CD11b (clone M1/70), CD11c (clone N418), F4/80 (clone BM8), CD44 (clone IM7), CD28 (clone 37.51), CCR7 (clone 4B12), CD69 (clone H1.2F3), CD62L (clone MEL-14), CD103 (clone 2E7), IFN-γ (clone XMG1.2), TNF-α (clone MP6-XT22), GZMB (clone QA16A02), and Tim-3 (clone F38-2E2) from BioLegend. Intracellular FOXP3 was stained using Foxp3/Transcription Factor Fixation/Permeabilization Concentrate and Diluent kit (00-5521-00, eBioscience) following the manufacturer’s instructions. For intracellular cytokine staining, 2 × 10^6^ cells were maintained in RPMI-1640 with 2% FBS and treated with 50 ng/mL PMA (P8139, Sigma-Aldrich), 2 μg/mL ionomycin (73722, STEMCELL Technologies) and 1.5 μl/ml Golgiplug (BDB555029, Thermo Fisher Scientific) for 4 hours, or with 10 μg/mL eGFP peptide _200_HYLSTQSAL_208_ (AS-65302, Anaspec) and Golgiplug for 6 hours. Cells were then stained with corresponding antibodies after fixation/permeabilization.

### Analysis of cytokine levels by ELISA.

ELISA kits for detection of IFN-α (42120-1), IFN-β (MIFNB0), IFN-γ (MIF00), TNF-α (MTA00B), IL-2 (M2000), IL-6 (M6000B), IL-10 (M1000B), IL-12 (M1270), and IL-23 (M2300) were purchased from R&D System. ELISA kits for detection of TGF-β (ab119557), IL-1α (ab199076), IL-1β (ab100705), and IL-4 (ab100710) were purchased from Abcam. Cytokine levels were determined using corresponding ELISA kits according to the manufacturer’s instructions.

### T cell killing assay.

T cell killing assay was performed as previously described (34). Human primary T cells (70024, STEMCELL Technologies) were maintained in RPMI-1640 with 10% FBS and 10 ng/mL of IL-2 (589102, BioLegend). For T cell expansion and activation, T cells were cultured in ImmunoCult-XF T Cell Expansion Medium (10981, STEMCELL Technologies) with 25 μL/mL of ImmunoCult Human CD3/CD28 T Cell Activator (10971, STEMCELL Technologies) and 10 ng/mL of IL-2 for 7 days. To analyze the killing effect of T cells, 3 × 10^5^ of tumor cells were cocultured with 3 × 10^6^ of activated T cells in DMEM with 10% FBS, 25 μL/mL of T cell activator and 10 ng/mL of IL-2 for 48 hours. Then LDH release assay was performed.

### Animal experiments.

All mice were obtained from Shanghai SLAC Laboratory Animals Co. Ltd (Shanghai, China) and maintained in the standard housing conditions (temperatures of 65°F–75°F with 40%–60% humidity and a 14-hour light/10-hour dark cycle) recommended by the Jackson Laboratory. For the orthotopic xenograft model, 2 × 10^6^ of MDA-MB-436 cells were injected into the mammary fat pads of 6-week-old female nude mice. Mice were orally administered olaparib (50 mg/kg) 4 times per week for 24 days. For orthotopic immunocompetent mouse model, 3 × 10^4^ of 4TO7 parental or stable cells were injected into the mammary fat pads of 6-week-old female BALB/c mice. Mice were orally administered olaparib (50 mg/kg) 5 times per week for 18 days. For CD8^+^ T cell depletion, intraperitoneal injection of 300 μg of CD8 antibody was performed on days 3, 7, 11, and 15 after tumor challenge. For PD-1 blockade and PARPi combination therapy, mice were treated with 50 mg/kg of olaparib 5 times per week or 100 μg/dose of PD-1 antibody (BE0146, BioXCell) twice a week, or the combination. For PanO2, MC38, Hepa-1-6, B16 tumor models, 5 × 10^4^ stable cells were injected subcutaneously into the right flank of C57BL/6 mice, and mice were orally administered olaparib (50 mg/kg) 5 times per week for 18 days. All in vivo experiments were conducted with 10 mice for each group.

### Statistics.

GraphPad Prism 9.0 was used for statistical analysis. All data are presented as the mean ± SD. The Shapiro-Wilk test and Brown–Forsythe test were used to assess normality and equal variances between group samples, respectively. When normality and equal variance were achieved between sample groups, 1-way ANOVA (followed by Dunnett’s correction), 2-way ANOVA (followed by Šídák’s correction), or unpaired 2-tailed *t* test were used. When normality of samples failed, the Kruskal–Wallis 1-way ANOVA (followed by Dunn’s correction) was used. When normality was achieved but equal variance failed, the Brown-Forsythe 1-way ANOVA (followed by Dunnett’s T3 correction) or unpaired 2-tailed *t* test with Welch’s correction were used. Fisher’s exact test and Pearson χ^2^ test were used for IHC analysis, and a log-rank test was used for survival analysis. Differences were considered statistically significant when the *P* value was less than 0.05.

### Study approval.

All animal studies were conducted in accordance with the Guide for the Care and Use of Laboratory Animals (Ministry of Health, China) and the protocol was reviewed and approved by the Animal Care and Use Committees of the Laboratory Animal Research Center at Xiangya Medical School of Central South University. All TNBC tissue samples were collected with written informed consent from patients and following the guidelines approved by the ethics boards of the Xiangya Hospital.

### Data availability.

Values for all data points shown in graphs are provided in the Supporting Data Values XLS file. The representative dot plot images for Figure 3C are reported as Supplemental data.

## Author contributions

J. Hou designed and conceived the study. J. Hou, XZ, and MH wrote the manuscript. J. Hou and SW performed experiments and analyzed data. CC analyzed the mouse GSDMC sequence. J. Huang and SZ provided patient tissue samples. MH and J. Hou supervised the entire project.

## Supplementary Material

Supplemental data

Supporting data values

## Figures and Tables

**Table 2:**
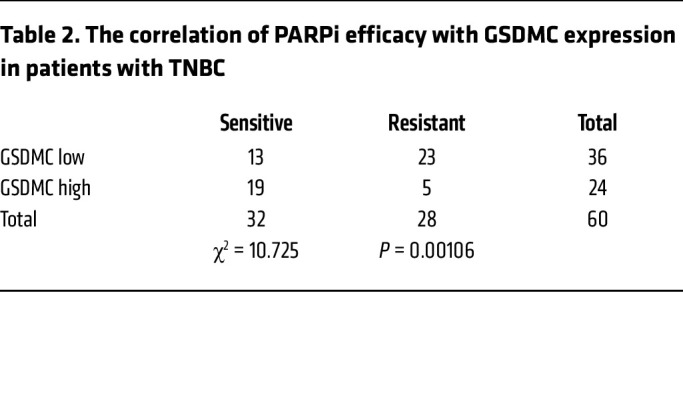
The correlation of PARPi efficacy with GSDMC expression in patients with TNBC

**Table 1:**
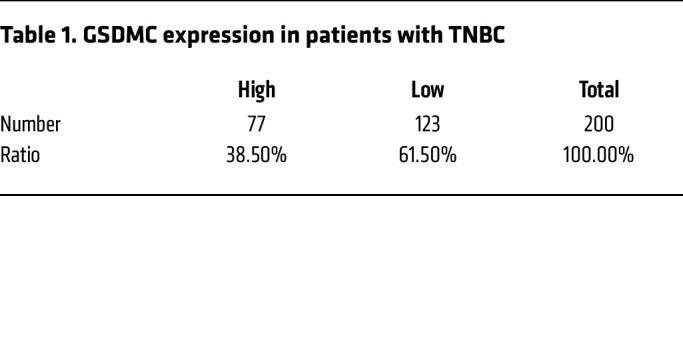
GSDMC expression in patients with TNBC

## Version 1 10/26/2023

In-Press Preview

## Version 2 01/02/2024

Electronic publication

## References

[1] Feng FY (2015). Chromatin to clinic: the molecular rationale for PARP1 inhibitor function. Mol Cell.

[2] LaFargue CJ (2019). Exploring and comparing adverse events between PARP inhibitors. Lancet Oncol.

[3] Lord CJ, Ashworth A (2017). PARP inhibitors: synthetic lethality in the clinic. Science.

[4] Pilié PG (2019). State-of-the-art strategies for targeting the DNA damage response in cancer. Nat Rev Clin Oncol.

[5] Murai J (2012). Trapping of PARP1 and PARP2 by clinical PARP inhibitors. Cancer Res.

[6] Meng XW (2014). Poly(ADP-ribose) polymerase inhibitors sensitize cancer cells to death receptor-mediated apoptosis by enhancing death receptor expression. J Biol Chem.

[7] Paldino E (2020). Modulation of inflammasome and pyroptosis by olaparib, a PARP-1 inhibitor, in the R6/2 mouse model of Huntington’s disease. Cells.

[8] Hou J (2020). PD-L1-mediated gasdermin C expression switches apoptosis to pyroptosis in cancer cells and facilitates tumour necrosis. Nat Cell Biol.

[9] Pantelidou C (2019). PARP inhibitor efficacy depends on CD8^+^ T-cell recruitment via intratumoral STING pathway activation in BRCA-deficient models of triple-negative breast cancer. Cancer Discov.

[10] Sen T (2019). Targeting DNA damage response promotes antitumor immunity through STING-mediated T-cell activation in small cell lung cancer. Cancer Discov.

[11] Kinjyo I, Adams SF (2020). PARP-inhibition induces cancer immunogenic cell death in response to high levels of interferon-gamma in the tumor microenvironment. Cancer Res.

[12] Zhou Z (2020). Granzyme A from cytotoxic lymphocytes cleaves GSDMB to trigger pyroptosis in target cells. Science.

[13] Zhang Z (2020). Gasdermin E suppresses tumour growth by activating anti-tumour immunity. Nature.

[14] Wang Q (2020). A bioorthogonal system reveals antitumour immune function of pyroptosis. Nature.

[15] Adrain C (2005). Molecular ordering of the caspase activation cascade initiated by the cytotoxic T lymphocyte/natural killer (CTL/NK) protease granzyme B. J Biol Chem.

[16] Galluzzi L (2017). Immunogenic cell death in cancer and infectious disease. Nat Rev Immunol.

[17] Hou J (2021). Molecular mechanisms and functions of pyroptosis in inflammation and antitumor immunity. Mol Cell.

[18] Ding J (2016). Pore-forming activity and structural autoinhibition of the gasdermin family. Nature.

[19] Wang Y (2017). Chemotherapy drugs induce pyroptosis through caspase-3 cleavage of a gasdermin. Nature.

[20] Jiao S (2017). PARP inhibitor upregulates PD-L1 expression and enhances cancer-associated immunosuppression. Clin Cancer Res.

[21] Stewart RA (2018). Development of PARP and immune-checkpoint inhibitor combinations. Cancer Res.

[22] Lampert EJ (2020). Combination of PARP inhibitor olaparib, and PD-L1 inhibitor durvalumab, in recurrent ovarian cancer: a proof-of-concept phase II study. Clin Cancer Res.

[23] Lee HH (2019). Removal of N-linked glycosylation enhances PD-L1 detection and predicts anti-PD-1/PD-L1 therapeutic efficacy. Cancer Cell.

[24] Ou-Yang F (2022). De-glycosylated membrane PD-L1 in tumor tissues as a biomarker for responsiveness to atezolizumab (Tecentriq) in advanced breast cancer patients. Am J Cancer Res.

[25] Tan G (2020). HMGB1 released from GSDME-mediated pyroptotic epithelial cells participates in the tumorigenesis of colitis-associated colorectal cancer through the ERK1/2 pathway. J Hematol Oncol.

[26] Erkes DA (2020). Mutant BRAF and MEK inhibitors regulate the tumor immune microenvironment via pyroptosis. Cancer Discov.

[27] Hanahan D, Weinberg RA (2011). Hallmarks of cancer: the next generation. Cell.

[28] Chu YY (2022). Targeting the ALK-CDK9-Tyr19 kinase cascade sensitizes ovarian and breast tumors to PARP inhibition via destabilization of the P-TEFb complex. Nat Cancer.

[29] Du Y (2016). Blocking c-Met-mediated PARP1 phosphorylation enhances anti-tumor effects of PARP inhibitors. Nat Med.

[30] Gao Y (2021). Nuclear translocation of the receptor tyrosine kinase c-MET reduces the treatment efficacies of olaparib and gemcitabine in pancreatic ductal adenocarcinoma cells. Am J Cancer Res.

[31] Han Y (2019). Synergism of PARP inhibitor fluzoparib (HS10160) and MET inhibitor HS10241 in breast and ovarian cancer cells. Am J Cancer Res.

[32] Dong Q (2019). EGFR and c-MET cooperate to enhance resistance to PARP inhibitors in hepatocellular carcinoma. Cancer Res.

[33] Chu YY (2020). Blocking c-Met and EGFR reverses acquired resistance of PARP inhibitors in triple-negative breast cancer. Am J Cancer Res.

[34] Lim SO (2016). Deubiquitination and stabilization of PD-L1 by CSN5. Cancer Cell.

