# Supplementary material for: Gasdermin C sensitizes tumor cells to PARP inhibitor therapy in cancer models
Source: J Clin Invest. 2024 Jan 2;134(1):e166841. doi: 10.1172/JCI166841 (PMC10760963; doi:10.1172/JCI166841)
Supplement: Supplemental data [file jci-134-166841-s185.pdf]

Supplementary Information

Gasdermin C sensitizes tumor cells to PARP inhibitor therapy in cancer models

Shuanglian Wang, Chiung-Wen Chang, Juan Huang, Shan Zeng, Xin Zhang, Mien-Chie Hung, Junwei Hou

Supplemental Figure 1

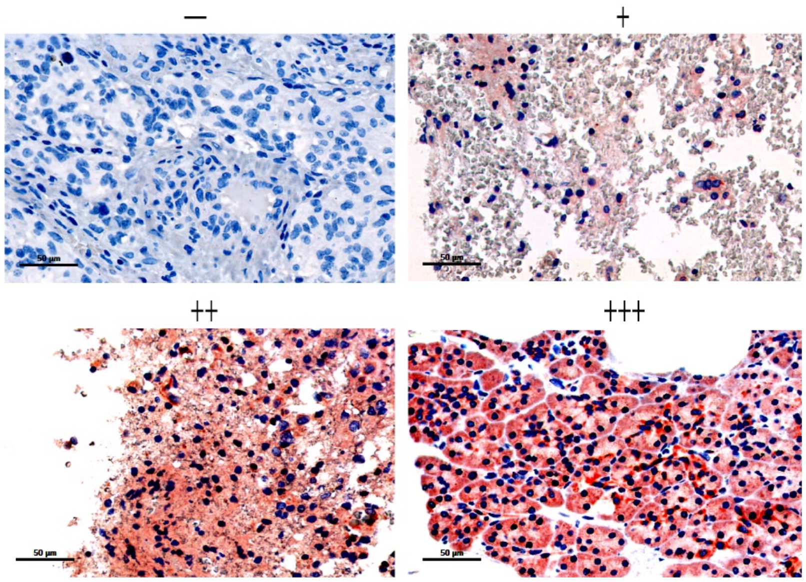

Supplemental Figure 1. Representative immunohistochemical (IHC) staining results for GSDMC in human TNBC tissues. Scale bar, 50 μm.

Supplemental Figure 2

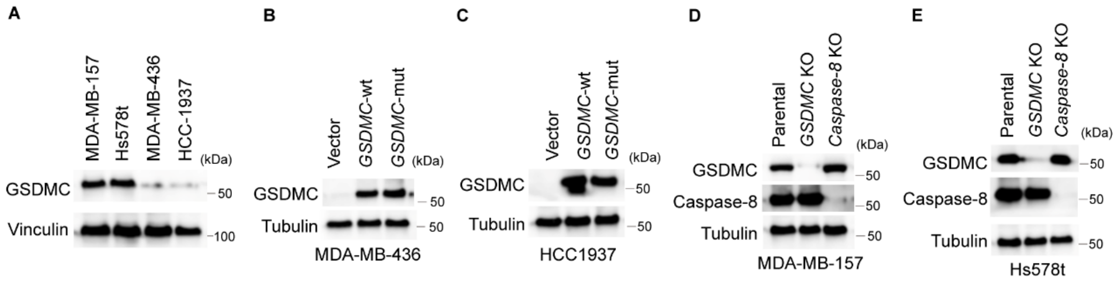

Supplemental Figure 2. GSDMC and caspase-8 expression in TNBC cells. (**A**) Endogenous GSDMC expression level in TNBC cells as indicated. (**B** and **C**) GSDMC expression level in MDA-MB-436 and HCC1937 stable cells harboring an empty vector (Vector) or expressing wild-type *GSDMC* (*GSDMC*-wt) or the D365A mutant (*GSDMC*-mut). (**D** and **E**) Establishment of MDA-MB-157and Hs578t stable cells with deletion of *GSDMC* or *caspase-8*.

Supplemental Figure 3

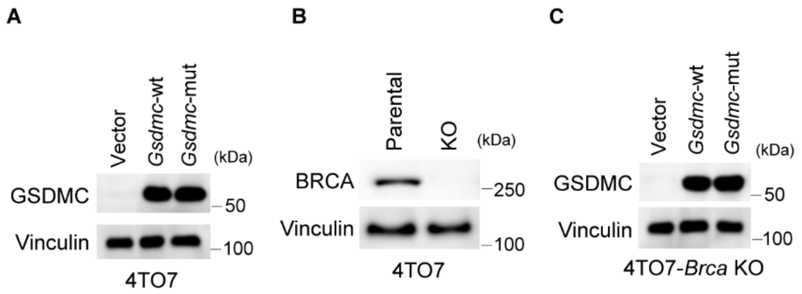

Supplemental Figure 3. Establishment of 4TO7 stable cells. (**A**) Enforced mouse *Gsdmc* expression in 4TO7 cells. Empty vector, vector; Wild-type mouse *Gsdmc*, *Gsdmc*-wt; The caspase-8 cleavage site D263A mutant, *Gsdmc*-mut. (**B**) Deletion of *Brca* gene in 4TO7 cells. KO, *Brca* knockout. (**C**) Ectopic expression of vector, *Gsdmc*-wt, and *Gsdmc*-mut in 4TO7-*Brca* KO cells.

Supplemental Figure 4

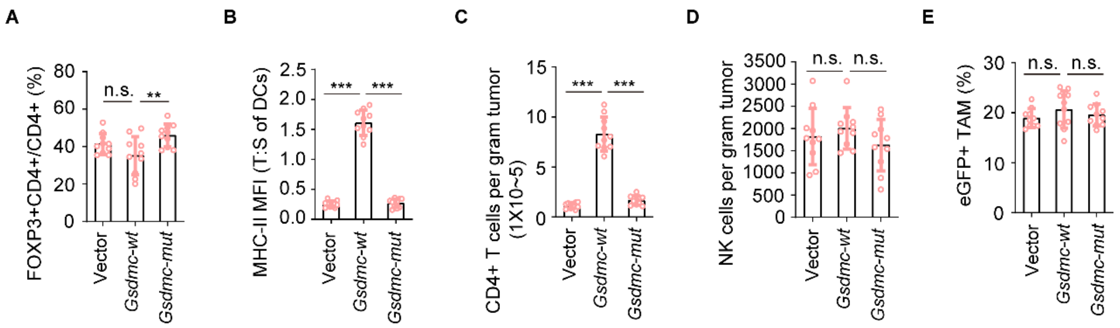

Supplemental Figure 4. Infiltrating immune cells in 4TO7-*Brca* KO tumor. (**A-D**) 4TO7-*Brca* KO cells stably expressing an empty vector (vector) or wild-type mouse *Gsdmc* (*Gsdmc*-wt) or the D263A mutant (*Gsdmc*-mut) were inoculated into the mammary fat pad of immunocompetent BALB/c mice (n = 10). Mice were orally administered olaparib (50 mg/kg) five times per week for 18 days. Percentage of Treg cells (**A**). Percentage of activated DC cells (**B**). Mean numbers of CD4+ T cells/gram of tumor tissue (**C**). Mean numbers of NK cells/gram of tumor tissue (**D**). (**E**) Ectopic expression of *eGFP* in the stable cells as indicated in (**A-D**). Then cells were inoculated into the mammary fat pad of immunocompetent BALB/c mice (n = 10). Mice were orally administered olaparib (50 mg/kg) five times per week for 18 days. Percentage of eGFP+ macrophages that have engulfed tumor cells. Data represent mean ± SD. 1-way ANOVA was used. **p < 0.01, ***p < 0.001. n.s., no significance.

Supplemental Figure 5

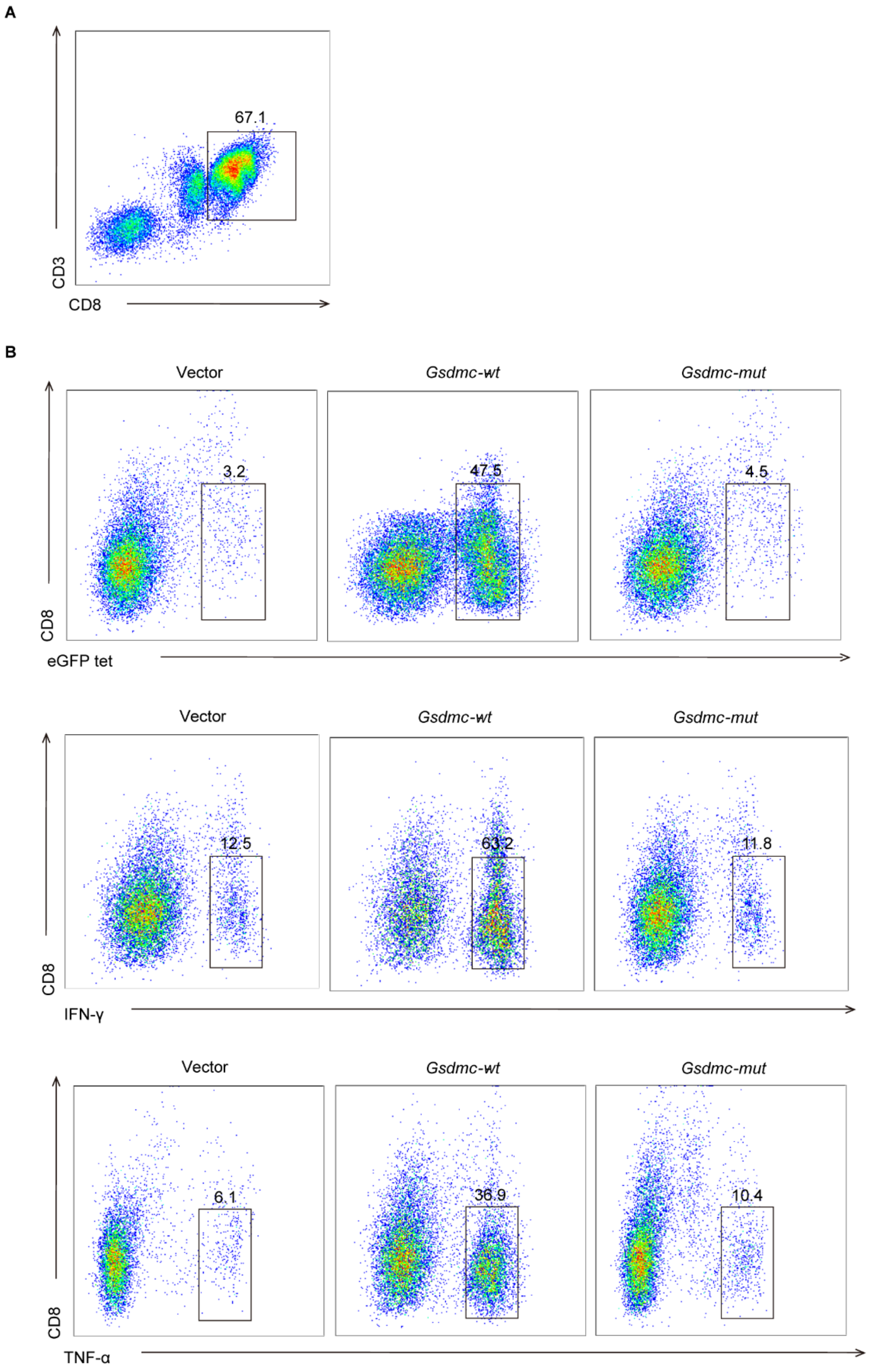

Supplemental Figure 5. Gating strategy and representative flow-cytometry plots for assessing tumour-infiltrating CD8+ T cells (A) or IFN-γ/TNF-α/eGFP tet-positive CD8+ T cells (B).

Supplemental Figure 6

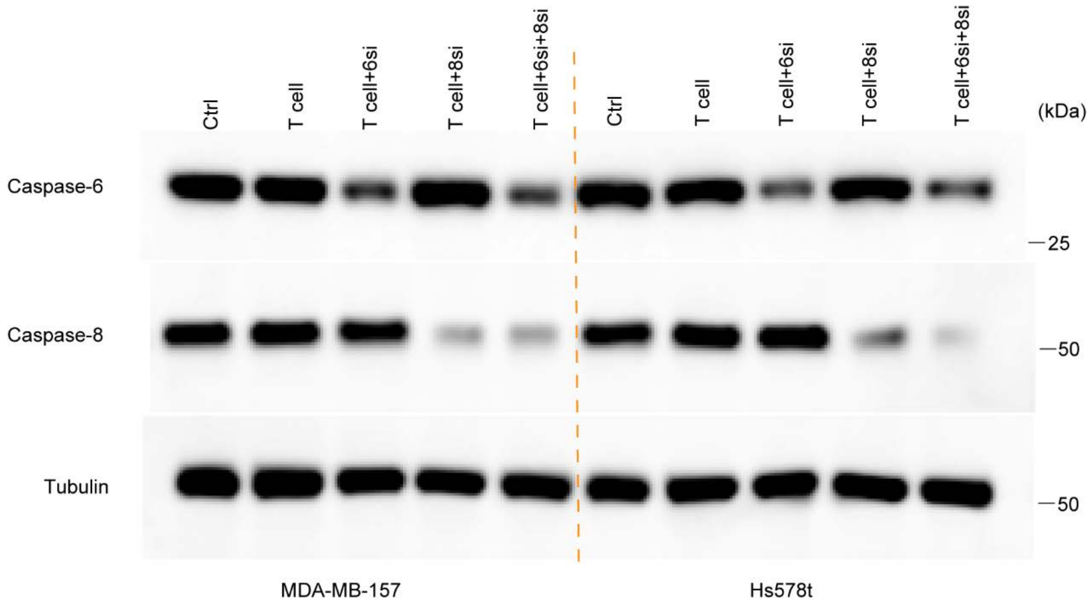

Supplemental Figure 6. Knockdown of caspase-6 and caspase-8 in MDA-MB-157 and Hs578t cells. Ctrl, control. 6si, caspase-6 siRNA treatment. 8si, caspase-8 siRNA treatment.

Supplemental Figure 7

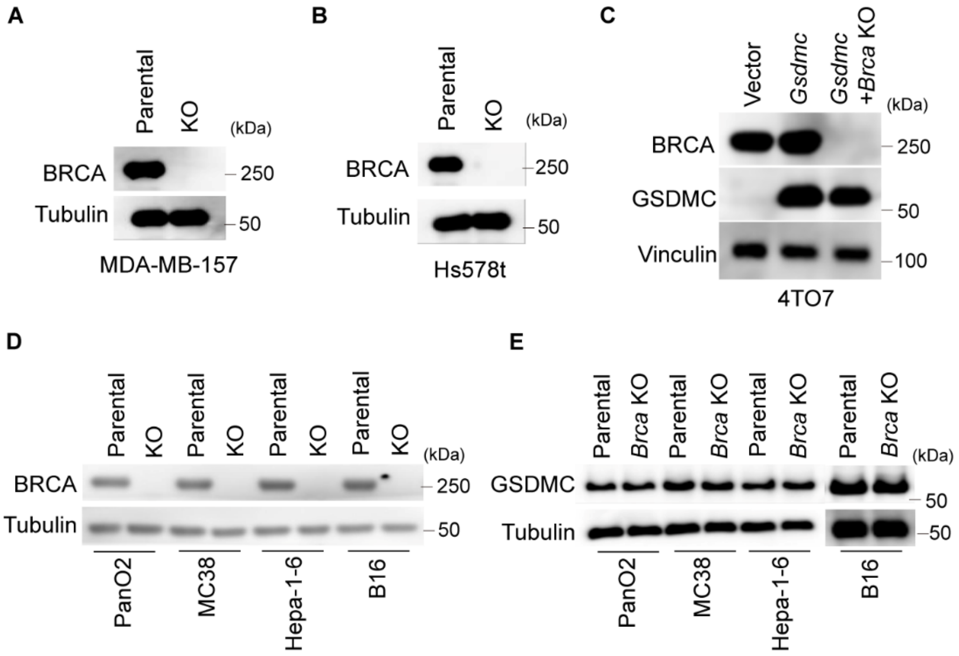

Supplemental Figure 7. Establishment of *BRCA* knockout and/or *GSDMC* overexpressing stable cells. (**A** and **B**) Knockout of *BRCA* gene in MDA-MB-157 (**A**) and Hs578t (**B**) cells. (**C**) Ectopic expression of *Gsdmc* and/or deletion of *Brca* in 4TO7cells. (**D**) Knockout of *Brca* gene in mouse cell lines as indicated. (**E**) Ectopic expression of *Gsdmc* in stable cells established in (**D**).

Supplemental Figure 8

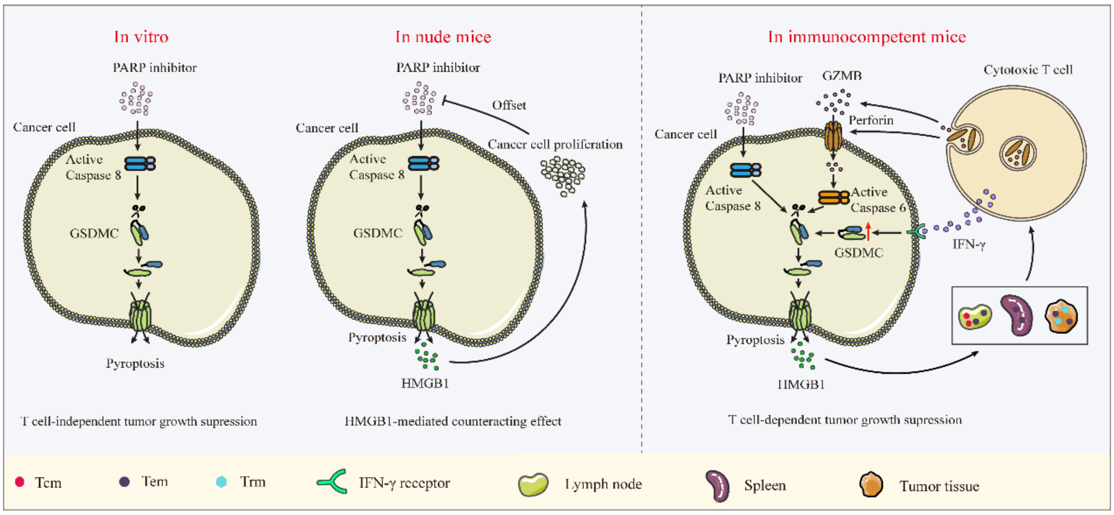

Supplemental Figure 8. A proposed model of GSDMC-mediated pyroptosis increasing PARPi sensitivity in cancers by enhancing antitumor immunity. PARPi induces caspase-8 cleavage of GSDMC to trigger CCP. The killing effect of PARPi is offset by pyroptotic cell-released HMGB1 through promoting cancer cell proliferation in nude mice. However, GSDMC-mediated CCP activates Tcm and Tem in lymph node, Tem in spleen, Tem and Trm in tumor microenvironment, and thus increases tumor infiltration and cytotoxicity of T cell. T cell-derived GZMB further triggers CCP by caspase-6 cleavage of GSDMC. Interestingly, IFN-γ that is generated from CCP-activated cytotoxic T cell upregulates GSDMC expression in cancer cells, which in turn enhances the cytotoxicity of PARPi and T cell. Taken together, GSDMC-mediated CCP kills tumor cells and augments antitumor immunity in an exacerbating feedback manner in response to PARPi treatment.

Supplemental Figure 9

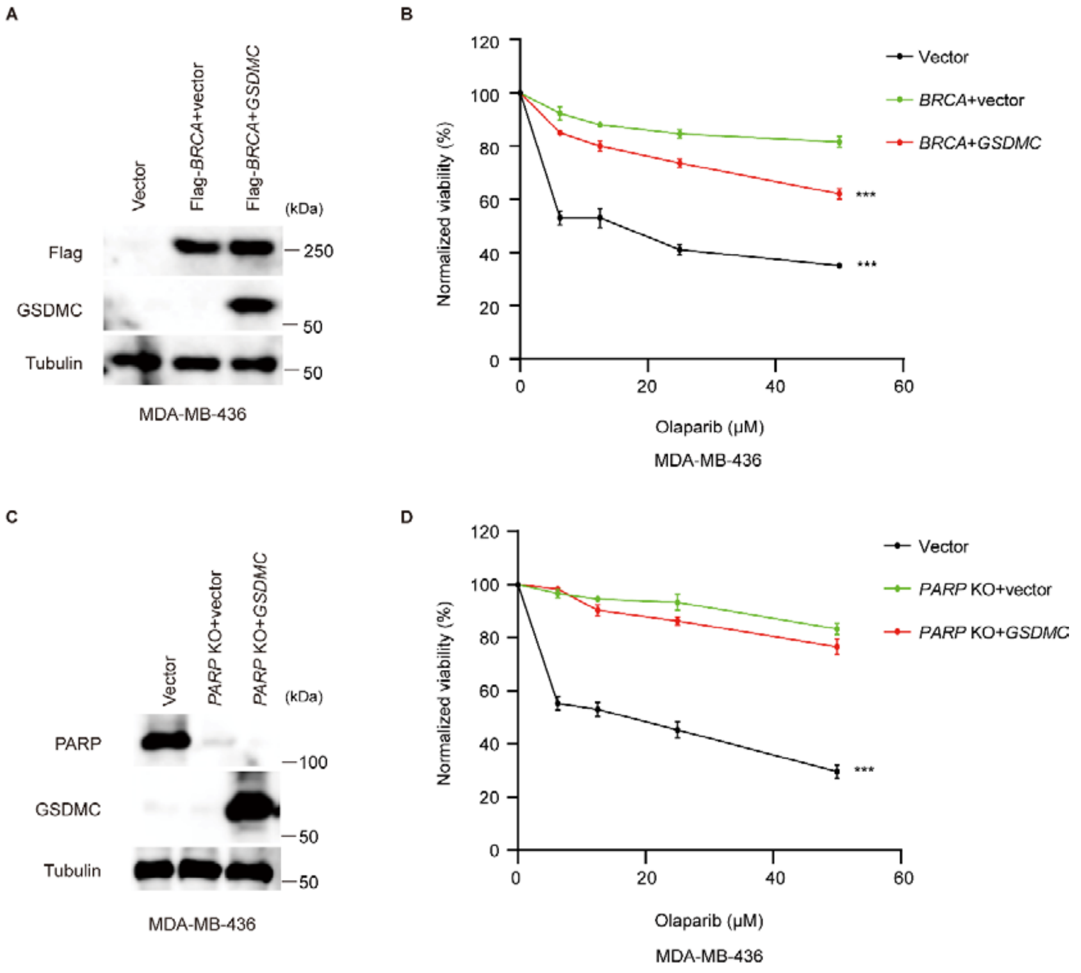

Supplemental Figure 9. The impact of *BRCA* reverse mutation and *PARP1* loss on GSDMC-mediated CCP. (**A**) Enforced expression of reverse-mutated *BRCA1* ($_{4065}$TCAAGAAGA$_{4073}$ deletion in *BRCA1* gene) in MDA-MB-436 cells with *GSDMC* overexpression. (**B**) Cells in A were treated with the indicated concentrations of olaparib for 72 h and subjected to a cell viability assay (n = 5). (**C**) Deletion of *PARP* in MDA-MB-436 cells with *GSDMC* overexpression. KO, knockout. (**D**) Cells in C were treated with the indicated concentrations of olaparib for 72 h and subjected to a cell viability assay (n = 5). Data represent mean ± SD. 1-way ANOVA was used. ***$p < 0.001$.

Supplemental Figure 10

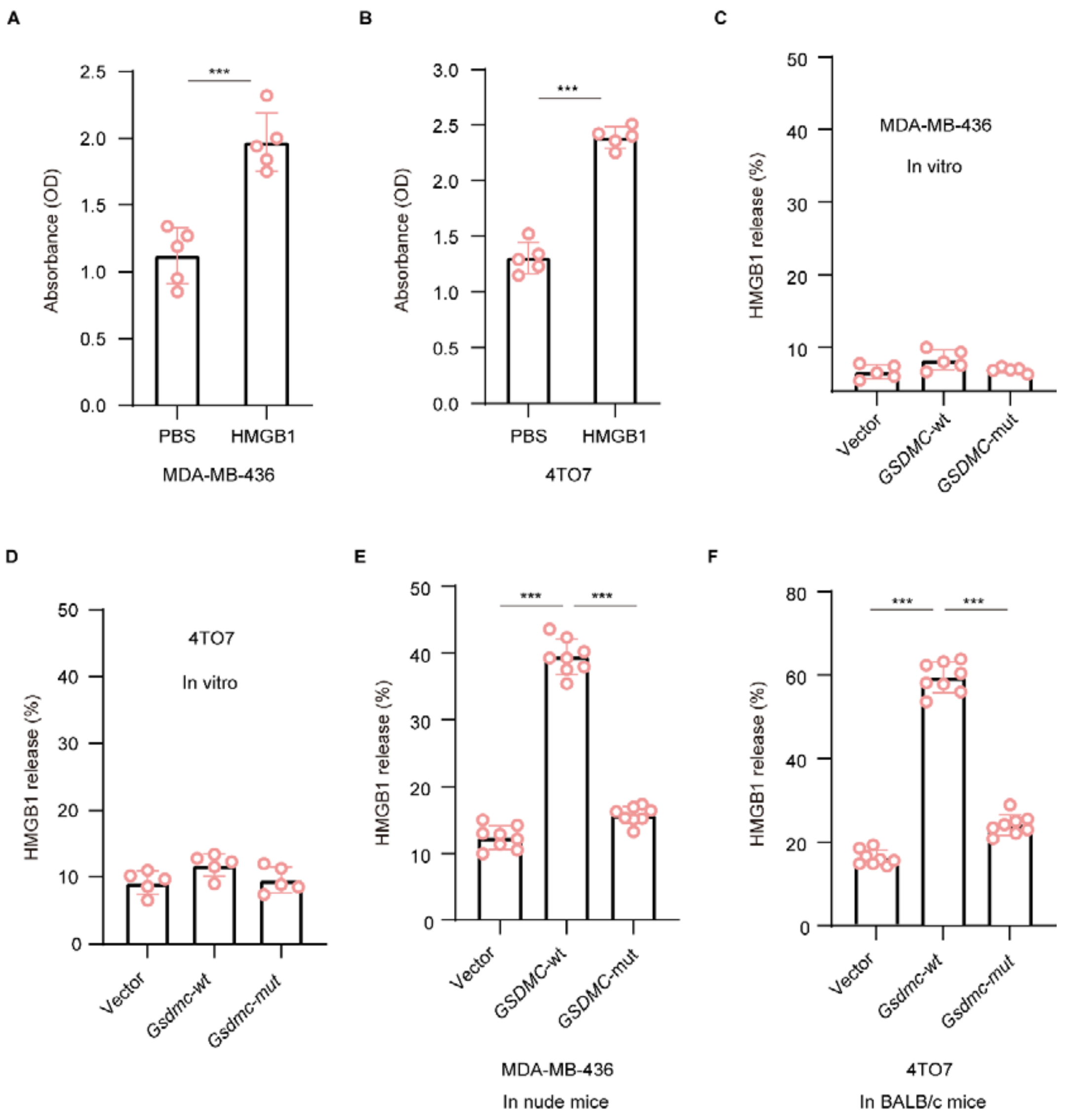

Supplemental Figure 10. The release of HMGB1 protein in vitro and in vivo in response to PARPi treatment. (**A** and **B**) CCK-8 assay to determine the proliferation of MDA-MB-436 (n = 5) (**A**) and 4TO7 (n = 5) (**B**) cells treated with HMGB1 protein (0.5 μg/mL). (**C** and **D**) HMGB1 release from MDA-MB-436 (**C**) and 4TO7 (**D**) stable cells as indicated under PARPi treatment in vitro (n = 5). (**E** and **F**) HMGB1 release in tumor slurry of MDA-MB-436 tumors as indicated in nude mice (n = 8) (**E**)

and of 4TO7 tumors as indicated in immunocompetent BALB/c mice (n = 8) (**F**). Data represent mean ± SD. Unpaired 2-tailed t test for **A**, **B**. 1-way ANOVA for **C-F**. ***$p < 0.001$.
